# What I Say is What I Get: Stronger Effects of Self-Generated vs. Cue-Induced Expectations in Event-Related Potentials

**DOI:** 10.3389/fpsyg.2012.00562

**Published:** 2012-12-14

**Authors:** Maike Kemper, Valentin J. Umbach, Sabine Schwager, Robert Gaschler, Peter A. Frensch, Birgit Stürmer

**Affiliations:** ^1^Department of Psychology, Humboldt-Universität zu BerlinBerlin, Germany

**Keywords:** self-generated expectations, cue-induced expectations, event-related brain potentials, N2, P3, lateralized readiness potential

## Abstract

Expectations regarding future events enable preparatory processes and allow for faster responses to expected stimuli compared to unexpected stimuli. Expectations can have internal sources or follow external cues. While many studies on expectation effects use some form of cueing, a direct comparison with self-generated expectations involving behavioral and psychophysiological measures is lacking. In the present study we compare cue-induced expectations with self-generated expectations that are both expressed verbally in a within-subjects design, measuring behavioral performance, and event-related brain potentials (ERPs). Response time benefits for expected stimuli are much larger when expectations are self-generated as compared to externally cued. Increased amplitudes in both the N2 and P3 components for violations of self-generated expectations suggest that this advantage can at least partially be ascribed to greater perceptual preparation. This goes along with a missing benefit for stimuli matching the expected response only and is mirrored in the lateralized readiness potential (LRP). Taken together, behavioral and ERP findings indicate that self-generated expectations lead to increased premotoric preparation compared to cue-induced expectations. Underlying cognitive or neuronal functional differences between these types of expectation remain a subject for future studies.

## Introduction

Expectations play a crucial role in action control. Research on effect-based action control has stressed that representations of anticipated action effects play a role when performing an action (e.g., Nattkemper et al., 2010). According to the ideo-motor principle (see Shin et al., 2010, for a recent review) the mental representation of an anticipated action effect triggers the action (similar to forward and inverse computational models of motor control, e.g., Wolpert and Ghahramani, 2000). For instance, the representation of an open drawer might help us to initiate the pulling action. By choosing actions according to the anticipated effects, people can gain intentional control over their behavior (e.g., Kunde, 2001; Pfister et al., 2010). They can consider expectations about upcoming action effects for choosing between actions depending on which effects they desire or not. As such, expectations about effects stem from goals of the actor. They might not be directly caused by current external stimulation, but rather be self-generated by integrating goals and past external stimulation. Interestingly, this view often does not directly translate to the methodology of experiments on the role of action effect anticipation in action control. For instance, the role of anticipated effects has been studied by presenting action effects additionally as subliminal stimuli (e.g., Kunde, 2004) or irrelevant flankers (e.g., Zießler and Nattkemper, 2002). One could argue that presenting to-be-expected effects as stimuli might trade experimental control against external validity, as such a situation is not closely resembling action preparation driven by self-generated expectations. Conceivably, intentional action control supposes self-generated expectations. These are likely to interact with stimulus-based preparation but are unlikely identical to this. For instance, according to Kunde et al. (2007) actors use anticipated action effects based on internal goals. Yet, stimuli have an important role in this view, too. They disambiguate situations as to whether or not an effect can be brought about by an action. As many actions only lead to the desired outcomes in highly specific contexts, the role of a stimulus is to signal that in the current context the link between expected effect and action is valid.

Taken together, this reasoning might suggest that the presumed equivalence between self-generated expectations and cue-induced expectations cannot be taken for granted. It is also conceivable that self-generated expectations differ from expectations that are directly triggered by external stimuli or cues. A similar distinction has been discussed with respect to internally triggered vs. externally cued task switching (Arrington and Logan, 2005). Differences between expectations based on external cues and internal sources are also conceivable given the long history of debates concerning motor patterns that are predominantly stimulus-triggered vs. predominantly driven by a response goal. For instance, the Baldwin–Titchener debate at the end of the nineteenth century (e.g., Baldwin, 1895; Titchener, 1895) centered around the question of whether or not response times (RTs) are regularly shorter when people concentrate on the response rather than on expecting the stimulus. An important insight of that debate was that people can apparently choose between different modes for controlling the same motor pattern.

In line with these precursors, recent results point to differences between intentional vs. reactive action. Surprisingly, a motor pattern already triggered by an internal goal is incompatible with the execution of the very same motor pattern in response to a stimulus presented while the intentional action is in preparation (e.g., Astor-Jack and Haggard, 2005; Pfister et al., 2012). If an internally prepared action is truncated by a stimulus that requires the same action that was intentionally prepared, RT costs result in comparison to a situation where the response could be executed without concurrent intentional preparation. The authors interpret their results as evidence for distinct action systems that are triggered either endogenously by intention or exogenously by an imperative stimulus. Presenting the stimulus during intentional action preparation therefore results in interference between both systems and delays the action. In line with these results, Herwig et al. (2007) have differentiated two types of action control modes, a stimulus-based action control mode and an intention-based action mode. Pfister et al. (2011) have shown that previously acquired action effect associations either impact performance or not, depending on which of these two modes is operating. One can of course debate what exactly differentiates the intention-based from stimulus-based action mode (e.g., Neuringer and Jensen, 2010), however, empirical data highlights that different paths to action do exist.

While our current study is inspired by recent work on effect-based action control, we focus on distinguishing between self-generated vs. cue-induced stimulus expectations. Such a focus is feasible given that theories on integration of perception and action (e.g., Hommel, 2009; Magen and Cohen, 2010) suggest that action effects and stimuli share the same representational basis. Studying self-generated vs. cue-induced expectations is driven by the conjecture that anticipating appropriate environmental conditions in order to prepare for efficient goal-directed actions is one of the core abilities of our neurocognitive system (e.g., Kunde et al., 2007). Anticipation, prediction, and expectancy are only some of the labels used to discuss such mechanisms (e.g., Sutton and Barto, 1981; Elsner and Hommel, 2001; Jentzsch and Sommer, 2002). Here we use the term expectation in a broad sense, encompassing both the process of expecting as well as the object of this process. Expectations can originate from prior experience, when events occurring with a high frequency in the past are expected to be more likely to occur again in the future (e.g., Fitts et al., 1963). Expectations may as well rest upon situational cues that provide advance information about upcoming events (e.g., Posner and Snyder, 1975). Whatever the source, performance is usually boosted when the expected event occurs, whereas unexpected events impair performance (e.g., Acosta, 1982).

Previous studies of expectation have often exclusively relied on the use of external cues (e.g., Shulman et al., 1999; Oswal et al., 2007). Cueing allows a more rigid experimental manipulation of the induced expectations as compared to a setup with self-generated expectations. However, before jumping to the conclusion that cueing should be used to study expectation in general, potential functional differences between endogenous and exogenous expectations should be scrutinized. To our knowledge, the only direct comparison of self-generated and cue-induced expectations was carried out by Acosta (1982). In a series of experiments he pitted predictions verbalized by participants against cues (words that announced a certain stimulus and were to be read aloud). As he included neutral expectations as a control, he could differentiate the facilitation of correct expectations from the cost of a wrong expectation. Furthermore, he manipulated the expectation-target interval and found effects of the interval duration in the prediction condition for both benefits of matches and costs of mismatches. Benefits increased with longer expectation-target intervals while costs were highest at the shortest intervals. The effects were generally much smaller in the cue condition. Mismatch costs were also highest at the shorter intervals while no significant benefits for matches of cue-induced expectations were found. In a second experiment he manipulated the number of the response alternatives by mapping more than one stimulus to a response. The expectation effect did not increase linearly with the number of alternative responses, indicating that the process responsible for expectation effects is not just a scaling effect in choosing between the possible alternatives to predict. Moreover, his findings suggested that expectation effects were bound to stimulus processing rather than to response processing. As multiple stimuli were mapped to the same response, an expectation concerning a stimulus could be violated while the response to be executed was the same that would have been appropriate in case of a stimulus matching the expectation. Responses in such trials were as slow as those to unexpected stimuli with a different response. This suggests that the expectation effect is not (solely) a part of response execution.

Comparing different behavioral effects of self-generated vs. cue-induced expectation, Acosta (1982) concluded that the types of expectation differed only in the magnitude of their effects but not qualitatively. It therefore appears expedient to study self-generated vs. cue-induced expectations with respect to their effects on action preparation including neural measures that are more independent of the overt responses and could better differentiate quantitative from qualitative effects. In the current study we aimed to replicate the behavioral findings of Acosta (1982), showing stronger effects of self-generated compared to cue-induced expectations. Moreover, we used event-related brain potentials (ERPs) to further distinguish the contribution of different cognitive processes to expectation effects in these two conditions. This includes potential differences between the two types of expectation prior to stimulus presentation. Qualitative differences in preparatory activity would be in accordance with theories that assume different routes to action (e.g., Astor-Jack and Haggard, 2005; Kunde et al., 2007; Pfister et al., 2011).

Explicit self-generated expectations about upcoming stimuli measured on a trial-by-trial basis (through verbalization) have not been a focus of recent research. To analyze the processes during the build-up of the expectations and response preparation, we used EEG recordings. There are two main questions we wanted to address with this study. First, do differences between the expectation types already exist prior to stimulus presentation? Second, which cognitive processes (perception, action selection, motor preparation) are influenced by expectation? More specifically, do self-generated expectations affect other processes than cue-induced expectations (qualitative differences between the expectation types) or affect the same processes but with a different magnitude (quantitative differences)?

We manipulated the type of expectation within-subjects. In the prediction condition participants had to verbally express their expectation regarding the upcoming stimulus, in the cue condition they had to read aloud a word naming the upcoming stimulus. Stimuli were simple shapes or colors. The task was then to react as fast as possible to the imperative stimulus with the right or left index finger. Since there were four stimuli, with two mapped to each finger, three types of matches or mismatches existed. First, for *stimulus matches* the expected (cued or predicted) stimulus matched the upcoming stimulus. Second, for *response matches* the expected stimulus did not match the upcoming stimulus but required the same response. Third, for *mismatches* the expected stimulus and the upcoming stimulus were different and did not require the same response either.

In addition, we included a manipulation of stimulus frequency. The two stimuli mapped to each finger were shown with different frequencies, at either 33 or 17% of all trials. Both hands had to respond equally often. The frequency manipulation was included to guide the participants’ predictions and to provide a measure indicating whether participants base their predictions on their experience (instead of random guessing). In a similar paradigm, Umbach et al. (2012) have shown that participants closely match their stimulus predictions to the observed frequencies. Even though expectations in their study were not valid in predicting the stimulus (similar to the current study) participants nonetheless used these expectations in preparing their responses.

The role of expectation in action preparation can be studied by comparing trials in which upcoming stimuli fulfill vs. do not fulfill expectations in behavioral measures (RTs and errors, e.g., Acosta, 1982) or with regard to effects in the brain that can for instance be measured by EEG (e.g., Matt et al., 1992; Jentzsch and Sommer, 2002). There are multiple processes that can lead to the expectation mismatch effects. It is possible that a correct expectation (a) facilitates the encoding of the stimulus, (b) the response selection, (c) response execution, or a combination of these. It is also possible that an expectation that does not match the stimulus delays one of these processes, or else that both – fulfilled and unfulfilled expectations – have opposing effects. Time differences in RTs and the latencies of the different ERPs which occur during the different stages prior to the response can help to show the stage(s) where the expectations exert their influence. ERP amplitudes can provide information about the magnitude of the involved processes in the different conditions.

### Contingent negative variation

To investigate whether there is a difference of cue-induced vs. self-generated expectation even before the stimulus is shown, we charted the contingent negative variation (CNV). This is a slow negative potential following an event cueing the upcoming target stimulus (inducing expectations in our case). The CNV develops in the cue-target interval and its amplitude is most pronounced directly before onset of the imperative stimulus. Depending on task demands, the late phase of the CNV reflects sensory, cognitive, or motor preparation (Damen and Brunia, 1994; Fan et al., 2007). Acosta (1982) has shown stronger RT effects in self-generated as compared to cue-induced expectations. A possible cause of this difference may be that the internal generation of expectations results in a larger amount of specific preparation that could, consequently, show up in a more pronounced CNV in the prediction condition.

### N2

The N2 is an ERP characterized by a larger amplitude in cases where the stimulus deviates in form or context from the prevailing stimulus (for a review, see Patel and Azzam, 2005). The N2 is also larger in response conflict trials as evoked by incongruent flanker or no-go trials (Kopp et al., 1996). Therefore, we explored whether mismatch between either kind of expectation and the upcoming target would result in an enlarged N2 amplitude. Larger interference effects in the N2 have been demonstrated in the Eriksen flanker task with a greater proportion of incongruent trials (Tillman and Wiens, 2011). As the interference effect on RTs was smaller in this condition, the N2 might reflect endogenous attention processes. If we assume that self-generated expectations have a stronger influence on preparatory processes (e.g., attention), the violation of an expectation might result in a larger N2 effect in the prediction condition compared to the cue condition.

### P3

Matt et al. (1992) and Jentzsch and Sommer (2002) differentiated between passive and active forms of expectations. While passive expectations automatically affect behavior, active expectations act in a rather controlled manner (Kahneman and Tversky, 1982). Matt and colleagues induced active expectations through instruction (“Expect stimulus repetitions!” “Expect stimulus alternations!”) in a blockwise manner. P3 amplitude as well as RTs revealed the higher order repetition effects typically found in simple reaction time tasks (stimulus repetitions benefit if they continue a run of repetitions, alternations if they continue a run of alternations). Importantly, the RT effect but not the P3 effect was modulated by the instructed expectation (expecting repetitions reduced the sequential effect for repetitions and increased that for alternations, and vice versa). This dissociation suggests that active and passive forms of expectation differentially affect processing stages involved in performing the task but might not show up in the P3.

However, operationalization of active and passive forms of expectation differed between Matt et al. (1992) and the current study. In contrast to their experimental approach, self-generated expectations in the current study were allowed to change on a trial-by-trial basis and were induced by stimulus frequency. Self-generated expectations might lead to stronger P3 effects as compared to cue-induced effects, because generating expectations internally trial-by-trial might lead to more pronounced processing of the expectation as compared to reading a cue. If one considers the relation of stimulus to expectation (rather than considering the stimulus alone), there are various possibilities for P3 effects. On the one hand, it is possible that the P3 relates to expectation by capitalizing on stimulus probability. In the current design, expectations often mismatch the actual stimuli. Even if a participant exclusively relies on the frequent stimulus, expectation matches are rare. Therefore, upon stimulus presentation, a P3 could follow in case of matches. On the other hand, P3 may reflect the accuracy of a concrete expectation on a single-trial basis rather than reflecting the past frequency of this expectation being fulfilled. In this case, a stimulus mismatching the expectation should elicit the higher P3 amplitude.

### Lateralized readiness potential

At the other end of the processing stream, the lateralized readiness potential (LRP) can be used to infer the role of response preparation in expectation effects (e.g., Jentzsch and Sommer, 2002). The LRP is a difference waveform that arises with a higher activity in the motor area of the brain hemisphere contralateral to the responding hand as compared to the ipsilateral hemisphere (Coles, 1989). The onset of the stimulus-locked LRP (S-LRP) provides a chronometric index of premotor processing stages (Leuthold et al., 1996) while onset differences in the response-locked LRP (LRP-R) indicate processing differences at late motor-related stages (Hackley and Valle-Inclán, 1998). Jentzsch and Sommer (2002) found that S-LRP was significantly influenced by the expectations, while the LRP-R was not. This shows that the instructed expectation influenced a process after early stimulus processing (as P3 was not affected in this study) but prior to the response initiation. Accordingly, we assumed expectation effects specifically on the S-LRP that should be particularly strong in case of self-generated expectations.

### Influences of stimulus frequency

While the main focus of our experiment lay on the comparison of cue-induced vs. self-generated expectations, the variation of stimulus frequency we applied also needs to be briefly summarized. Obviously the experimenter has little control over expectations self-generated by participants. By varying stimulus frequency it should be possible to partly shape self-generated expectations and to be able to explore how self-generated expectations accommodate to the task environment (see Umbach et al., 2012). Specifically, reliance on stimulus frequency can be considered a sign of subjective validity of the self-generated expectations that participants are asked to verbalize. Furthermore, the more frequent stimuli should lead to faster responses as compared to less frequent stimuli. Potential effects of stimulus frequency may in part be independent of expectation match effects in the current trial (compare Jiménez and Méndez, 2012). Conceivably, stimulus frequency leads to a sustained effect more similar to the passive form of expectation that Matt et al. (1992) found reflected in the P3. We expected larger P3 and N2 components for infrequent as compared to frequent stimuli.

Furthermore, the CNV is seen to reflect preparatory processes and the amplitude is, for example, modulated by cue validity (if the upcoming stimulus is specified with different probabilities). CNV amplitude is larger the more valid the cues (and thus, the more expected the stimuli) are (Scheibe et al., 2009). We therefore expect a larger CNV for the expectations of frequent stimuli since these are more likely to be fulfilled (33 vs. 17% validity).

## Materials and Methods

### Participants

Eighteen participants (four men) with a mean age of 24.7 years took part in the experiment. All Participants were right-handed and had normal or corrected-to-normal vision. The participants were either psychology students at Humboldt-Universität zu Berlin and participated in exchange for course credit or received a compensation of €20 for the experiment with a duration of approximately two and a half hours. Participants gave their informed consent prior to the experiment.

### Apparatus and software

The Experiment was programmed with MathWorks MATLAB and the Psychophysics Toolbox (Brainard, 1997; Pelli, 1997) and presented on a Windows computer. The participants’ expectations were recorded using a table microphone and played to the experimenter who coded the predictions on a separate computer outside the EEG booth. Error feedback after erroneous responses was given via tabletop speakers.

### Stimulus material and experimental manipulation

The stimuli were either simple shapes (house, star, cross, and gate) or colored circles (blue, red, green, and yellow) presented on a computer monitor with a light gray background. Stimuli were 22 mm in diameter, corresponding to a visual angle of about 2.1° at a viewing distance of approximately 60 cm. The experiment consisted of two parts: a cue-induced (cue condition) and a self-generated expectation variant (prediction condition). One of these parts was performed with colors as stimuli and the other with shapes. The order of the expectation variants as well as the assignment to the two types of stimuli was randomized across participants.

In trials of the cue condition, the participants were presented with the one-syllable word for one of the stimuli, which they were instructed to read aloud (the German equivalents for house, star, cross, and gate in the shape condition, or the German equivalents for blue, red, green, and yellow in the color condition). In the prediction condition they saw a prompt – the German equivalent for “color?” or “shape?” – to which they should respond by naming the stimulus they expected to appear in the current trial. Thus, verbal output consisted of the same words in both expectation conditions.

**Figure 1:**
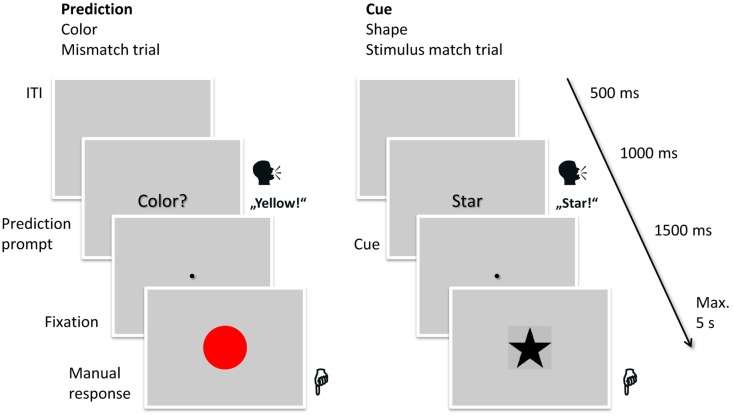
**Task used in the experiment**. On each trial, participants either had to read aloud the cue (in this case “star”) or to verbalize their prediction for the upcoming stimulus (in this case “yellow”). After 1,000 ms the fixation point appeared on screen and after another 1,500 ms the stimulus appeared on screen (in this case red in the prediction trial, signifying a mismatch, and a star in the cued trial, signifying a match). The participants manually responded to the stimulus by pressing one of two keys. The next trial started 500 ms after the response. The mapping of the stimulus types (shape, color) to the expectation conditions and the order of the expectation conditions was balanced over participants.

Participants had to react to the stimuli by pressing one of two buttons with either the left or the right index finger. Depending on the current type of stimuli, each button corresponded to two forms or two colors. The mapping was randomized, shown before the experiment and was trained during two training blocks. Of the two stimuli per hand, one was presented in one out of three trials (33% = frequent stimuli) and the others in one out of six (17% = infrequent stimuli; half as often as the frequent stimuli), together resulting in the same frequency (50%) of responses with each hand. The order in which the stimuli were presented was randomized. In the cue condition, the frequency of the cues was matched to the frequency of the stimuli (cues for frequent stimuli were shown in 33% of the trials, cues for infrequent stimuli in 17%). The task is shown in Figure 1.

### Task procedure and instructions

After being introduced to the lab and the experimental procedure, participants provided their consent to participate and were seated in a one person lab room and prepared for the EEG measurements. Next a detailed explanation of the task in the following experiment and the stimulus-response mapping was presented on the screen and also explained by the experimenter. Instructions explained the course of the trials, the response mappings and the request to relax the mouth as soon as possible after pronouncing the expectation (i.e., as soon as the fixation point presented in response to the registration of the expectation). This was included to ensure minimized muscle artifacts in the EEG measurements.

The first training block of eight trials followed. After that, a shorter version of the instructions was presented and any questions that arose during the first training block could be clarified with the experimenter. This was followed by another training block, after which the experimenter left the room and the participant could start the experiment by pressing a button. The experiment consisted of two parts, each containing five blocks of 108 trials. The length of the breaks between the blocks could be controlled by the participants. The second half of the experiment contained a switching of the stimuli and expectation condition. There were again two training blocks of eight trials each preceded by instructions explaining the new task. To minimize mistakes, the stimulus-response mapping was shown before every block. If the wrong button was pressed an acoustic error feedback was given; it was also given when no button had been pressed within 5 s following stimulus presentation.

Each trial in the experimental blocks began with the presentation of either the cue or the prompt for the expectation in the middle of the screen. After 1,000 ms, the fixation point was shown at the same point. After another 1,500 ms, the stimulus was shown until a button press was registered or for 5 s if no reaction followed during that time. This was followed by an intertrial interval of 500 ms before the next trial started with the presentation of a cue or prediction prompt.

At the end of the session participants were asked to estimate the frequency of the characteristic stimulus values.

### Electrophysiological recordings

Recordings were made from Ag/AgCl electrodes mounted in an electrode cap (Easy-Cap) at 25 scalp positions (FP1, FP2, F3, F4, F7, F8, C3, C4, T7, T8, P3, P4, P7, P8, O7, O8, O1, O2, FPz, Fz, FCz, Cz, CPz, Pz, Oz) according to the extended 10–20 system. AFz served as ground electrode. In addition, external electrodes were used for recording the vertical and horizontal electrooculogram as well as for the mastoids. The electrodes were referenced to the linked mastoids. Electrode impedance was kept below 5 kΩ. The EEG was recorded with a sampling rate of 1,000 Hz and no online filters were applied. Blink artifacts were corrected semi-automatically by independent component analysis (ICA) using the ICA algorithm integrated in the BrainVision Analyzer 2.0 (BrainProducts GmbH). Offline, the continuous EEG was separated into individual trials with 300 ms pre- and 2,700 ms post-cue epochs (cue-locked data, in the prediction condition they were locked to the presentation of the prompt), and 200 ms pre- and 800 ms post-stimulus epochs (stimulus-locked data), and with 1,000 ms pre- and 200 ms post-response epochs (response-locked data).

### Data analysis

For data analysis, only trials with correct key presses were considered. For the CNV, the cue-locked segments were averaged according to the expectation condition (cue vs. prediction) and frequency condition (expectation corresponded to frequent or infrequent stimulus) and 30 Hz low-pass filtered. For the statistical analysis the difference between the mean voltage around the visual potential of the fixation point (1,400–1,200 ms prior to stimulus presentation) and the mean voltage 200 ms before the stimulus onset at electrode Cz was used with the baseline 200 ms before the onset of the cue or the prediction prompt. For the N2 and P3, the stimulus-locked segments were averaged according to the expectation conditions (cue vs. prediction) and match types (mismatch, response match, and stimulus match) and 30 Hz low-pass filtered (Butterworth, slope 12 dB/oct). The N2 amplitude was the mean amplitude measured at Fz between 250 and 350 ms after stimulus onset. P3 latency was measured as the time of the positive maximum at the Pz electrode during the time range of 250–550 ms following stimulus onset. The P3 amplitude was measured as the mean amplitude measured at Pz between 250 and 550 ms after stimulus onset. For both N2 and P3 the baseline was taken during a 200 ms pre-stimulus interval.

For the LRP, EEG was 5 Hz low-pass filtered (Butterworth, slope 12 dB/oct). The LRP was derived by computing difference waves for the C3 and the C4 electrodes between the electrode contralateral to the corresponding hand in a given trial and the ipsilateral electrode. Then the two types of difference waves (C3–C4 for right-hand response trials and C4–C3 for left-hand response trials) were averaged within each of the experimental conditions (cue mismatch, cue response match, cue stimulus match, prediction mismatch, prediction response match, prediction stimulus match). LRP onsets were analyzed using a jackknife-based procedure for factorial designs (Ulrich and Miller, 2001). Eighteen different grand average LRPs for each of the experimental conditions were computed by omitting the ERP data of one participant from each grand average. This allowed to measure the usually noisy LRP onsets much more precisely than on a single participant. LRP onsets were measured in the waveform of each grand average and submitted to an ANOVA with *F*-values corrected as *F*_c_ = *F*/(*n* − 1)^2^, with *F*_c_ as the corrected *F*-value and *n* as the number of participants. S-LRP onsets were measured with a 200 ms pre-stimulus baseline and LRP-R with a 100 ms baseline, starting 100 ms after the responses were made. As Miller et al. (1998) recommended, we used a relative criterion of 50% of the maximal LRP amplitude during the recording epoch for determining the LRP onsets for both the S- and the R-locked LRPs.

### Speech artifacts and verbalization latency

The participants were asked to verbalize their expectation as soon as the prompt or cue was shown and to relax their facial muscles again as soon as the fixation point was shown. The EEG data acquired during the time of speech was not analyzed. The earliest data points used in the analysis were in the Cz amplitude (CNV), starting 100 ms after the presentation of the fixation point, which should render enough time for artifacts from muscles involved in the prior speech production to subside. Visual inspection of the microphone recordings showed activations in the frequency range of speech primarily prior to the presentation of the fixation point. In addition, the stimulus types and their mapping to the expectation condition and frequencies were randomized; thus their verbalization should not have been able to systematically influence any EEG measurements. Furthermore, participants were instructed to use the relatively long interval between the prompt or cue and the fixation point for blinking if necessary.

Analyzing processing differences with chronometric measures (as comparing ERP latencies) presumes equivalent starting points of the processes of interest. In our case it is assumed that possible preparatory processes start with the verbalization of either the prediction or the cue, respectively. Possibly, however, it is harder to generate a prediction than to read a cue. If, because of this, predictions are verbalized later than cues that have simply to be read aloud, preparation, on the one hand, may start later in the prediction condition and, on the other hand, the distance in time between the verbalization and the imperative stimulus would be shorter for predictions than for cues. Both influences would make a comparison of the time courses of the prediction and cue conditions problematic. Being aware of these difficulties we conducted a behavioral pilot study with the same materials that allowed a precise measurement of voice onset times. Moreover, anticipating possible differences in verbalization latency, we locked the time of stimulus presentation in this pilot study to voice onset time rather than using a fixed interval between prompt/cue and stimulus as in the main study reported here. The stricter controlled pilot study revealed the same behavioral effects of expectation as the EEG study. Importantly, we found no difference in verbalization latency between predictions and cues (though the different standard deviations may mirror a processing difference between producing one and the same word as a prediction or by reading)^1^ and decided for a fixed interval between prompt/cue and stimulus in the main study in order to avoid problems with incompatibilities between speech recognition and precise EEG recording.

## Results

### Exclusion of data

Training blocks were not analyzed. Furthermore, error trials were excluded from the RT and EEG analyses. Trials in which the participants had not reacted after 5 s were counted as error trials. According to this criterion 2.6% of all trials were excluded. Response time analyses were based on medians per participant and condition. Due to the experimental design, roughly twice as many mismatch trials went into the analysis compared to response matches and stimulus matches; this proportion was similar for both expectation conditions^2^. Predictions were matched by the correct stimulus in 25.9% of trials while cues were valid in 27.7%.

### Behavioral data

**Figure 2:**
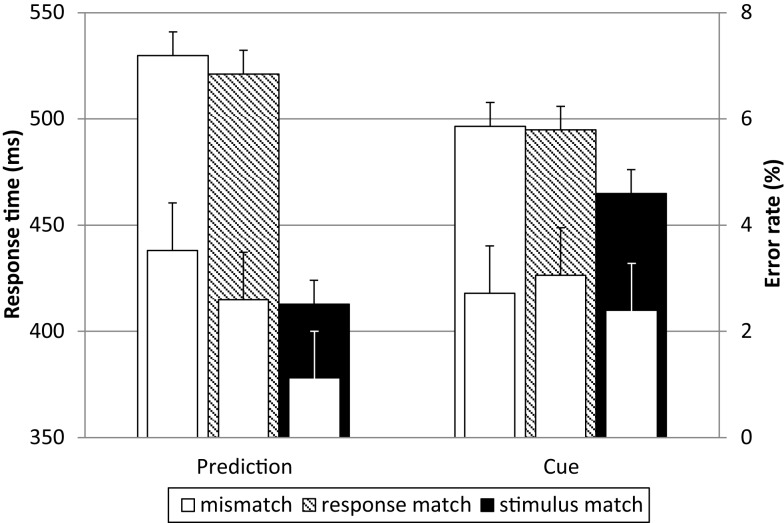
**Response times and error rates**. Response times (outer bars) exhibit an effect of match (with faster responses to stimulus matches than to response matches and mismatches) and an interaction with expectation condition (with a stronger effect of match in the self-generated predictions, left). The same pattern is visible in the error rates (inner bars). There were less errors made in the match trials, thus the effect in response times cannot be explained by a speed-accuracy trade-off. Error bars represent confidence intervals (95%) for repeated-measures designs according to Loftus and Masson (1994) and Jarmasz and Hollands (2009).

Response times and error rates can be seen in Figure 2. RT was on average 72 ms longer for mismatches than for stimulus matches. This slowing was about the same for response match and complete mismatch trials. Match trials were also more accurate than mismatch trials. The advantage of stimulus matches was larger for the prediction condition (Figure 2, left; 113 ms) than for the cue condition (Figure 2, right; 31 ms). Additionally, the RTs were 17 ms shorter for the frequent stimuli compared to infrequent stimuli. A three-way repeated-measures ANOVA with expectation condition, match and frequency as within-subjects factors on the median RTs rendered a significant main effect of frequency, *F*(1, 17) = 32.96, *p* < 0.001, $\eta_{p}^{2}=0.66$, and of match, *F*(2, 34) = 316.38, *p* < 0.001, $\eta_{p}^{2}=0.95$; there was no main effect of expectation condition, *F*(1, 17) = 0.06, ns. Importantly, there was a significant interaction of match and expectation condition, *F*(2, 34) = 36.78, *p* < 0.001, $\eta_{p}^{2}=0.68$, with a larger difference between the two types of mismatch and the stimulus match for the prediction condition than for the cue condition. *T*-tests revealed that for both expectation conditions there was no significant difference between mismatch and response match [both *t*(17) < 1.46, ns], while the stimulus match was significantly faster than both [all *t*(17) > 6.69, *p* < 0.001, all *d* > 3.38]. The effect of match on the error rates was in the same direction, *F*(2, 34) = 7.13, *p* = 0.003, $\eta_{p}^{2}=0.30$, with less errors for stimulus matches as compared to mismatches. The effects can therefore not be explained by a speed-accuracy trade-off.

The frequency manipulation was reflected in the prediction behavior, as participants predicted the more frequent stimuli on a larger proportion of trials, χ^2^(1) = 7.39, *p* = 0.007. The *post hoc* estimates of stimulus occurrence in % made by the participants also provide a good approximation of the actual frequencies, with the frequent stimuli at 59%, and the infrequent stimuli at 41% (for comparison, real presentation frequencies: 66 and 33%, respectively).

### Contingent negative variation

The CNV was neither influenced by the expectation condition nor by the frequency. A repeated-measures ANOVA for the influence of frequency and expectation condition revealed no main effect of expectation condition, *F*(1, 17) = 1.29, ns, or of frequency, *F*(1, 17) = 1.64, ns, and no interaction, *F*(1, 17) = 0.92, ns.

### N2

**Figure 3:**
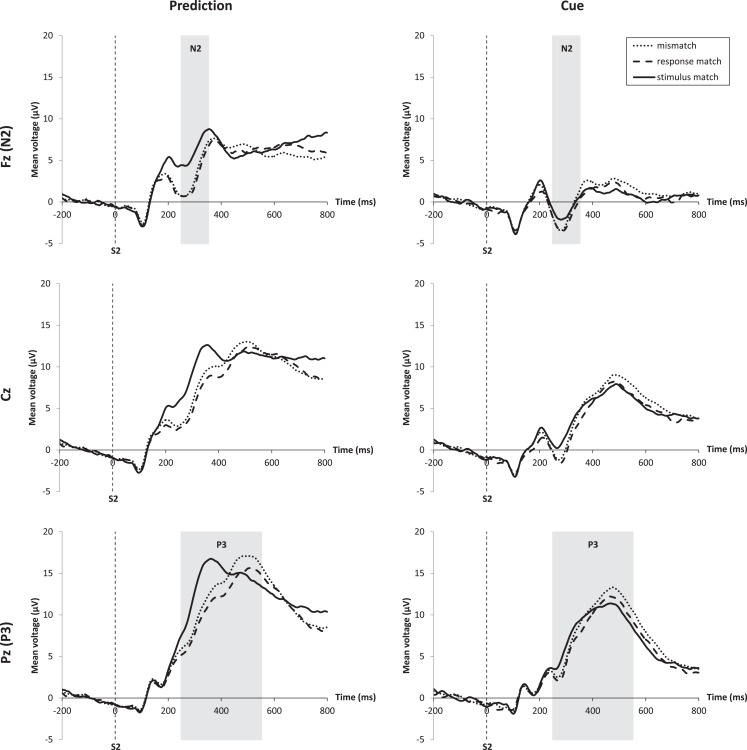
**ERPs at midline electrodes Fz, Cz, and Pz time-locked to stimulus onset**. Prediction condition is shown on the left, cue condition on the right. For each electrode the different waveforms for the three match types are shown. Analysis windows for N2 and P3 components are highlighted in gray. Stimulus matches are marked by the solid line, response matches by the dashed line, and mismatches by the dotted line. The interaction of match and expectation condition can best be seen at the Fz electrode for the N2 and at the Pz electrode for the P3.

Figure 3 (top) shows the N2 for the prediction and the cue condition at electrode Fz. The N2 amplitude was larger for the cue condition than for the prediction condition, and in both expectation conditions the N2 was larger for mismatches and response matches than for stimulus matches. The amplitude difference of response matches and mismatches compared to stimulus matches was larger for the prediction than for the cue condition. A repeated-measures ANOVA for the effects of match type and expectation condition on the mean amplitude of the N2 measured at Fz between 250 and 350 ms revealed a main effect for match, *F*(2, 34) = 15.52, *p* < 0.001, $\eta_{p}^{2}=0.48$ and a main effect for expectation condition, *F*(1, 17) = 39.14, *p* < 0.001, $\eta_{p}^{2}=0.70$. The interaction was based on a larger amplitude difference between the different match types for the prediction condition compared to the cue condition, *F*(2, 34) = 6.79, *p* = 0.003, $\eta_{p}^{2}=0.29$. A three-way repeated-measures ANOVA that also included the influence of frequency on the N2 peak amplitude rendered no main effect of frequency, *F*(1, 17) < 0.01, ns.

### P3

The P3 (Figure 3, bottom) had a larger amplitude for predictions compared to cues and for mismatches compared to stimulus and response matches. In the cue condition the full stimulus match exhibited the smallest P3 amplitude, with a higher amplitude for response matches and the highest amplitude for mismatches. In the prediction condition the pattern was more complex, with stimulus matches showing a much shorter peak latency of the P3 compared to all other conditions. A repeated-measures ANOVA for the effects of match type and expectation condition on the mean amplitude of the P3 revealed a main effect for match, *F*(2, 34) = 14.16, *p* < 0.001, $\eta_{p}^{2}=0.45$, a main effect for expectation condition, *F*(1, 17) = 16.23, *p* < 0.001, $\eta_{p}^{2}=0.49$, and a significant interaction, *F*(2, 34) = 6.83, *p* < 0.003, $\eta_{p}^{2}=0.29$. A three-way repeated-measures ANOVA that also included the influence of frequency on the P3 mean amplitude rendered no effect of frequency, *F*(1, 17) = 0.23, ns. There was a significant effect of match on the peak latency, *F*(2, 34) = 17.20, *p* < 0.001, $\eta_{p}^{2}=0.50$. A *t*-test revealed that this was due to the earlier P3 for stimulus matches in the prediction condition. The P3 for stimulus matches in the prediction condition began on average 85 ms earlier than for mismatches, *t*(17) = 5.57, *p* < 0.001, *d* = 2.70.

### Lateralized readiness potential

**Figure 4:**
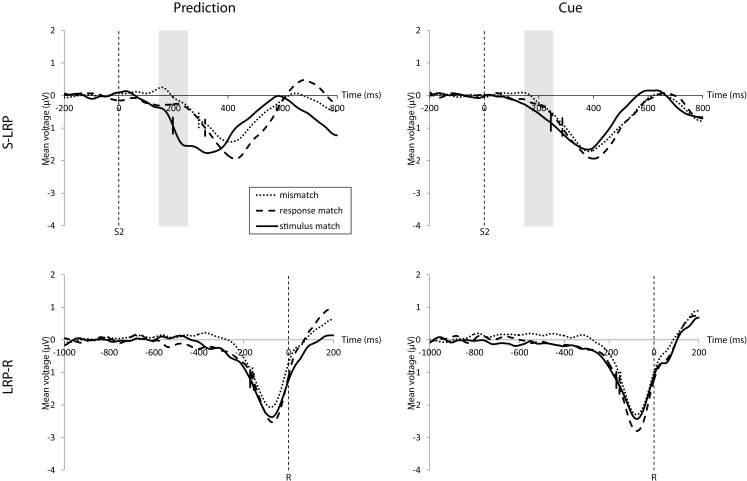
**Top: stimulus-locked LRP waveforms for the prediction condition (left) and cue condition (right)**. There was an earlier S-LRP onset for stimulus matches than for mismatches and response matches. (Onsets are marked by the short vertical lines intersecting the waveforms.) This onset difference was, in trend, larger for the prediction condition. Although the response match S-LRP onset is as late as for mismatches, they differ in their amplitude before S-LRP onset (50% of the maximum amplitude) in the time interval 150–250 ms following stimulus onset (highlighted in gray). The response match amplitude rises in the correct direction as with the stimulus match and is significantly higher than the mismatch amplitude, but only in the prediction condition. Bottom: response-locked LRP waveforms for the prediction condition (left) and cue condition (right). There is only a significant effect of match with an earlier LRP-R onset for stimulus matches compared to response matches and mismatches.

The onset of the S-LRP was earlier for stimulus matches than for response matches and mismatches, mirroring the RT results (Figure 4, top). A repeated-measures ANOVA for the influence of match and expectation condition on the S-LRP onset rendered a main effect of match, *F*(2, 34) = 24.33, *p* < 0.001, $\eta_{p}^{2}=0.59$, but not of expectation condition. There was a trend toward an interaction, *F*(2, 34) = 2.58, *p* = 0.090, $\eta_{p}^{2}=0.13$, with a larger difference between the S-LRP onset latency for the stimulus match compared to the response match and mismatch in the prediction condition compared to the cue condition.

As can be seen in Figure 4 (top) there was an early rise of the response match S-LRP (especially in the prediction condition) which then soon aligned with the mismatch S-LRP. According to this visual inspection we also analyzed the average S-LRP amplitude 150–250 ms after stimulus onset. A repeated-measures ANOVA for the influence of match and expectation condition on the S-LRP amplitude 150–250 ms after stimulus onset revealed a main effect of match, *F*(2, 34) = 19.44, *p* < 0.001, $\eta_{p}^{2}=0.53$, but not of expectation condition. There was a significant interaction of expectation condition and stimulus match condition, *F*(2, 34) = 3.92, *p* = 0.029, $\eta_{p}^{2}=0.19$. The average S-LRP amplitude in the prediction condition in this interval was 0.29 μV higher for response matches than for mismatches, *t*(17) = 2.20, *p* = 0.042, *d* = 1.06 but there was no such difference in the cue condition, *t*(17) = 0.65, ns. Even though it was not reflected in the response time this finding indicates an early correct motoric activation for response matches in the prediction condition.

The onset latency of the LRP-R was influenced only by match, *F*(2, 34) = 5.21, *p* = 0.011, $\eta_{p}^{2}=0.24$ but not by the expectation condition; there was no interaction (Figure 4, bottom).

## Discussion

The aim of the present study was to shed some light on the basic processes that underlie the effects of expectation on the control of performance. We were especially interested in distinguishing between the consequences of self-generated expectations (predictions) vs. cue-induced expectations. On each trial participants verbalized an expectation prior to stimulus onset in a two-choice discrimination task. The expectation was either freely generated by the participants (prediction) or specified by an external cue (a word denoting the discriminating stimulus feature). Our results suggest that when investigating effects of explicit expectation one should be aware of possible differences between internally and externally triggered anticipation processes: predictions showed stronger behavioral effects and stronger effects on most ERP components after stimulus presentation that are related to expectation. The two types of expectation showed different aftereffects once a matching or mismatching stimulus was presented. Predictions, therefore, differed substantially from cue-induced expectations.

Direct comparisons of behavioral and neuronal indicators between expectations induced by cues vs. self-generated expectations have been lacking so far. With respect to behavioral differences between the two types of expectation we replicated Acosta (1982). RTs were slower when the stimulus did not match the expectation as compared to a match. This difference was larger in the prediction than in the cue condition. Moreover, as in Acosta’s study, we found no benefit of response match trials over complete mismatch trials, suggesting that the expectation exerts its influence before response preparation. The results of error rates reflected RTs, contradicting a speed-accuracy trade-off. Additionally, as a consequence of the frequency manipulation in our experiment, participants also responded faster to the more frequent stimuli.

In the following we shall first discuss the relevant aspects of the ERP results on self-generated vs. cue-induced expectations. We shall then discuss how type of expectation might relate to similar distinctions in other aspects of action control.

### Stronger ERP effects for predictions vs. cues

The CNV did not reveal any differences between predictions and cues. If at all, differences in the cue-target interval between both conditions showed up in an early time window starting 450 ms after cue onset. This was, however, the time window comprising the speech artifacts. Furthermore, participants were instructed that blinks should be synchronized with speaking aloud. Although the time window of this cue-related positive deflection resembled those found in task switching paradigms (e.g., Nicholson et al., 2005; Li et al., 2012) we refrain from further interpretation until this positivity is replicated in a design excluding artifacts.

In the ERPs related to stimulus processing we found differences with respect to expectation match that were modulated by the source of expectation (prediction vs. cue). The N2 amplitude for response match and complete mismatch trials was larger than for stimulus match trials, and this difference was significantly larger in the prediction condition. The N2 has been reported to be larger for incompatibly cued stimuli (Kopp et al., 1996) and interpreted as reflecting cognitive control functions concerning incorrect response preparations. Thus, our results might reflect the need to control the prepared incorrect responses for stimulus mismatch trials. However, in case of a response match the response associated with the unexpected stimulus is correct in our experiment. Our finding of equal N2 amplitudes for response match and complete mismatch trials indicates that the control processes are triggered by the pure stimulus mismatch. This corresponds to the view that interprets the N2 as a sign of mismatch or conflict detection (e.g., Folstein and Van Petten, 2008; Nigbur et al., 2011). Our data suggests that the effect is elicited by the stimulus violating the expectation rather than by the response associated with a different stimulus than the one presented.

Expectation effects on the N2 are larger in the prediction condition. As the probability of a violation of the expectation was comparable for the prediction and the cue conditions it is unlikely that the match effect in the N2 mirrors just the probability of conflict. This finding corresponds to the view that preparing for a self-generated prediction involves endogenous attention processes to a greater degree as preparing for a cued stimulus. Furthermore, the N2 amplitude was generally higher in the cue condition. Though this main effect does not relate to our hypotheses, one might speculate that it possibly also reflects the “expectation mode” (self-generated vs. externally triggered). Presumably, expectations were weaker in the cue condition so that stimuli were generally “less expected” as compared to the prediction condition. This corresponds to the smaller expectation effects we found for the cue condition in the behavioral data and the other ERPs.

We obtained an interaction of match and expectation condition for the P3 amplitude. While usually higher P3 amplitudes have been found for infrequent stimuli (Fabiani et al., 1987), we were able to demonstrate a frequency-independent influence of subjective expectation on the P3. Our results differ from those of Jentzsch and Sommer, 2002, who did not find an influence of explicit expectation on the P3. A possible reason for this discrepancy may lie in methodological differences. In contrast to Jentzsch and Sommer, 2002; see also Matt et al., 1992), we allowed expectations to fluctuate on a trial-by-trial basis instead of manipulating them by instruction at a block-level. Inducing an expectation at the beginning of a block of trials might lead to a situation where this expectation is implemented for action preparation early on and afterward might be effective in action preparation on lower levels of representation while no longer being strongly represented as an expectation proper (compare e.g., Wenke et al., 2009, for a similar argument with respect to the implementation of instructed stimulus-response links). Furthermore our experimental approach differed from the one in the above studies in that we required participants to generate explicit expectations themselves instead of being asked to hold a specific expectation given by instructions. As a consequence, the design of the present study might have been more sensitive to detecting small effects on P3 amplitudes. Concluding from our data, we suggest that explicit self-generated expectation indeed affects early stimulus processing stages, even stronger so than cue-induced expectations.

There was a much earlier P3 peak for stimulus matches as compared to mismatches in the prediction condition. Though the component was similar in its form to the other experimental conditions, conceivably, some kind of signal of prediction success or affirmation might have played a role if the self-generated expectation proved to be correct. Usually, the latency of the P3 peak reflects the time of uncertainty resolution. Sutton et al. (1967) showed this for match trials in an experiment with explicit self-generated expectations about upcoming auditory stimuli (either single or double clicks). They analyzed match trials in which single clicks were expected. The P3 latency depended on the latency of the possible (unexpected) second click. In the conditions with earlier second clicks the P3 was also earlier because the uncertainty about whether the expectation matched could be resolved earlier. This does not explain why in our study the P3 is so much earlier for stimulus matches only in the prediction condition, while in the cue condition the P3 is as late for stimulus matches as for mismatches. In the cue condition, uncertainty regarding the correctness of preparation should be resolved similarly early as in the prediction condition. However, in accordance with the idea that self-generated expectations result in more preparation than cue-induced expectations, a stronger impact of uncertainty resolution in the prediction condition seems plausible. We looked at the scalp distribution for this component in order to check if there is an additional process responsible for the latency difference, but the distribution did not differ from the distributions around the P3 for the other conditions.

Furthermore, we found no frequency effect for the N2 or P3. Even though frequency affected RTs, these effects do not seem to stem from the processes involved in the generation of the N2 or P3. In contrast to our hypothesis and the results from Jentzsch and Sommer (2002), the more passive form of expectation generated by the stimulus frequency had no effect on the ERPs. This could be due to the relatively small frequency differences of the four stimuli. As the expectations for the more frequent stimuli in our experiment happened to be matched by the stimulus more often than for the infrequent stimuli, an effect of frequency or an interaction of frequency and condition could also have been expected to influence the CNV. Expectation validity has been shown to affect CNV amplitude (Scheibe et al., 2009). However, there were no effects of the frequency manipulation on the CNV in our data, perhaps due to the relatively small differences in stimulus frequency that resulted in equally small differences in expectation validity. Although two of the four possible stimuli were shown twice as often as the other two, the absolute difference in validity between frequent and infrequent stimuli amounted to only 17% (as compared to 25% differences and an overall higher validity, 50 vs. 75 and 100%, in Scheibe et al., 2009).

The LRP results only partially reflect our predictions. As expected, the S-LRP onset reflected the RT results for the different match types, showing that these effects are the result of premotoric processing stages. The interaction with the influence of the expectation condition only approached significance. In contrast to our hypothesis and the results of Jentzsch and Sommer (2002) there was a significant effect of match type on the LRP-R onset, similar to the S-LRP onset and the RT, with an earlier onset for stimulus matches than for the two mismatch types. That is, motor preparation started earlier in those cases with fast response selection. The expectation condition, however, did not affect motor preparation as measured by the LRP-R.

Response matches did not differ from complete mismatches in behavioral performance. Although response matches call for the same response as indicated by the cue or prediction, we did not find any benefit compared to complete mismatches. This finding suggests that response preparation depends on the imperative stimulus. Similarly, the N2 and P3 amplitude did not differ between response matches and mismatches whereas response matches differed significantly from stimulus matches in N2 and P3 amplitude measures. The facilitation of stimulus matches is reflected in the S-LRP onset and can, therefore, be attributed to perceptual and/or central parts of the preparation process. There was no difference between response matches and complete mismatches in the S-LRP and the LRP-R onset was similarly late for response match and mismatch. This is partly in line with what the theory of event coding (TEC; Hommel, 2009) would predict. Event codes are abstract codes encompassing features of perceived stimuli and (to be) produced actions. According to TEC, stimulus and response features are integrated into one event code. Event codes might be formed and retrieved both during prediction/cue processing and when the stimulus is presented and responded to (compare e.g., Wenke et al., 2007). Connecting and disconnecting features in an event code takes processing time. Thus, if we assume that explicit expectation provides some form of “preparative” event code, response matches, and complete mismatches should take longer than stimulus matches, in which all links set up by the expectation can be kept. This prediction is met by our data. However, TEC further predicts that complete mismatches are faster than response matches because a new event code is formed instead of disconnecting old and connecting new features as in the case of a response match (in a response match trial the predicted response has to be kept, but in combination with another stimulus). This prediction is not met because complete mismatches behaviorally do not differ significantly from response matches, and, in tendency, are rather slower than response matches.

Overall, S-LRP results mostly reflected behavioral performance. However, with self-generated predictions, both stimulus and response matches lead to an initial rise in the S-LRP, indicating an activation of the corresponding response. In the later course a faster rise for stimulus matches results in the S-LRP passing the onset threshold (defined at 50% of the peak amplitude) much earlier, while response matches do not pass this threshold before mismatches. This pattern suggests a preactivation of the correct response that was then inhibited due to the reevaluation after a different stimulus was shown. Presumably, inhibition seems to commence in response matches as soon as the mismatch between expected and presented stimulus is detected. This is interesting with regard to the role of stimuli in goal-directed action that Kunde et al. (2007) offer. They suggest that actions are generally goal-oriented and stimuli primarily serve to disambiguate between two variants: (1) a specific effect can be expected to follow an action in the current context, or (2) a goal is likely unattainable in the current context. Even simple actions such as button presses or operations of switches can have different effects depending on context factors. Presumably, the early S-LRP in response matches is indicating that action preparation, turning the expectation into an action goal, is no longer fostered (or even inhibited) once the stimulus signals a mismatch with the expectation.

### Differentiating types of expectation

We suggest that it is necessary to differentiate between self-generated and cue-induced expectations. This might be informative for research proposing similar distinctions with respect to other aspects of action control. For instance, in research on effect-based action control the role of action mode (free choice vs. stimulus-driven) in the acquisition (e.g., Herwig et al., 2007; Herwig and Waszak, 2012; Janczyk et al., 2012) or application (Pfister et al., 2011; Gaschler and Nattkemper, submitted) of action effect associations is under current debate. We suggest that effect anticipation might have an especially strong impact on action control if it is based on expectations about effects that stem from goals of the actor rather than being directly caused by current external stimulation. Expectations that are generated internally by integrating goals and past external stimulation might be represented more strongly as compared to cue-induced expectations, as the former need to be shielded against competing external stimulation (compare e.g., Dreisbach and Haider, 2008). When relying on cues that are present on each trial, a strong representation is not established as it is not necessary (compare e.g., Ballard et al., 1995).

We explain our results by a difference between self-generated and cue-induced expectations. A reviewer suggested an alternative account according to which the response time and ERP differences might be based on just one kind of expectation that plays out differently in these two experimental conditions. For instance, one could assume that the participant’s expectation is in most cases validly reflected in the prediction condition. Thus, in most trials the participant would be expecting exactly what she or he verbally indicates. In contrast, a randomly presented cue might mirror the expectation on just some of the trials. While the cue suggests the expectation of a specific stimulus, the participant might not always follow this suggestion and often expect a different stimulus instead. By this account, expectation effects in the cueing condition might be as strong as in the prediction condition for the subset of trials in which participants expect what the cue suggests. It would be even conceivable that in this subset of trials of the cueing condition expectation effects might be stronger than those of the prediction condition, as potentially cues and internally generated predictions could be combined. However, as there is possibly a substantial proportion of trials in which participants do not follow the cue, one could expect that effects are on average smaller in the cueing condition as compared to the prediction condition. Though our experiment was not designed to test this alternative account, we analyzed reaction time data to evaluate this idea. According to the above view there should be no (or even a reversed) difference between the cueing and the prediction condition in the subset of trials in which there was likely a match between cue and internally generated expectation. This should be the case for the fastest 10% of match trials in the cueing condition. Percentile analyses did not support this conjecture. The 10% fastest match trials in the cue condition were *slower* than the 10% fastest match trials in the prediction condition [Δ = 27.39 ms; *t*(17) = 2.77, *p* = 0.013].

A second possibility to address this concern is to scrutinize the influence of stimulus lag on the match effect in the cue condition. A typical fallacy often underlying predictions is the tendency to increasingly expect a stimulus alternation after longer runs of repetitions, also known as the “gambler’s fallacy” (Ayton and Fischer, 2004). If a cue-independent internally generated expectation is effective in the cueing condition, a stimulus should be increasingly expected the longer it has not been presented. Indeed, in our sample the mean prediction probability for a stimulus increased from 16% when it had been presented two trials before to 30% when the last presentation was five or more trials back. The probability to predict a first-order repetition was on average 25%. All contrasts between the prediction probabilities for a stimulus presented at lag 1 (repetition prediction) to lag 5 or more were statistically significant. So, the predictions of our participants seem to reflect a mixture of a “gambler’s fallacy”-like alternation bias and a first-order repetition bias. Therefore, if the cue matches a stimulus that has not been presented for several trials, the likelihood for the cue matching the “real” expectation should be highest. Consequently, one would expect the largest match effect at the longest lag of trials. We reanalyzed RTs of stimulus match and complete mismatch trials (there were not enough data points in some cells for response matches) of the cue condition. We found an effect for match, *F*(1, 17) = 38.75, *p* < 0.001, $\eta_{p}^{2}=0.70$, with no differences between lags [interaction match × lag: *F*(1, 17) = 1.43, *p* = 0.232], while RTs generally increase over lags for match and mismatch trials (main effect of lag: *F*(1, 17) = 8.88, *p* < 0.001, $\eta_{p}^{2}=0.34$). In the case of a stimulus repetition the effect tended to be larger (41 ms), rather than smaller, compared to the effect at longer lags (22, 20, 25, and 20 ms, for lags 2, 3, 4, and more than 4, respectively). Currently, our data does not support the view that there is only one kind of expectation effective in both the cueing and prediction condition. Rather, the data suggests that expectation in the cueing condition is different from expectation in the prediction condition. As these *post hoc* analyses provide only preliminary arguments, the task to disentangle the interactions between internal and externally motivated expectation remains open to future research.

One can further argue that self-generated expectations can not be controlled experimentally to the same extent as cue-induced expectations. Yet we suggest that it is warranted to (also) use self-generated expectations for studying effects of expectation on goal-directed action. Research on task switching has witnessed a similar case where presumably external validity and experimental control have to be balanced. It could be shown that a voluntarily initiated task choice results in different behavioral effects as compared with the situation where the task set to be implemented is triggered by a cue: voluntary task switches lead to much smaller task switching costs than cued task switches (Arrington and Logan, 2005). Thus, not only in the preparation of simple actions but also at the superordinate level of task sets there are differences between self-initiated and externally triggered processes. Participants in the Arrington and Logan (2005) study were instructed to choose freely between two possible tasks (with about the same frequency and in an approximately random manner). Thus, they decided on a task to prepare for, or, to put it differently, they expected to execute the chosen task as soon as the stimulus appeared (cf. Kunde et al., 2007). Accordingly, after being cued, they prepared to execute the task given by the cue. This situation, therefore, is similar to the approach of the current study: performance differences are observed as a consequence of preparation determined by internal or external sources. However, it is not clear if the differences are based on qualitative differences between internally or externally initiated task preparation processes, or if it may already be the source of expectation generation (i.e., before any preparation starts) that affects the consecutive task processes.

The findings from voluntary task switching suggest that the two paths to action might already differ prior to stimulus presentation. Accordingly, expectations prior to stimulus presentation may vary and differently affect action preparation depending on whether they are cue-induced or self-generated. Moreover, the idea of stimulus-based and intention-based action control modes (e.g., Herwig et al., 2007) can be mapped to what is (not) necessary to build-up explicit stimulus expectations in cueing vs. self-generation: while cues can potentially act as rather automatic triggers for a specific expectation (e.g., Bargh and Chartrand, 1999), the requirement to generate predictions can only be fulfilled intentionally (compare e.g., Jahanshahi et al., 2006). As expectations are a part of the action it seems plausible that participants are more likely to be in an intention-based mode if they generate expectations themselves. Moreover, expected or unexpected stimuli in this context represent feedback (i.e., action effects) to the expectations, and the contingency between expectations and stimuli should impact performance to a larger extent if it is acquired in an intention-based mode (Pfister et al., 2011). This could explain the performance differences between prediction and cue trials in Acosta’s (1982) and our study.

The difference between self-generated and cue-induced expectations and their role in action control requires further study. We have demonstrated that these types of expectation differ in a situation in which both are explicitly verbalized using the same words as output (naming the predicted differentiating stimulus feature vs. reading the cue word of this feature). A study trying to generalize the different expectation effects beyond this specific verbal task seems promising. Furthermore, it is necessary to test accounts of how and why self-generated and cue-induced expectations differ. As of yet, it is not clear whether the two types of expectation differ qualitatively or quantitatively. Self-generated expectations might either show stronger and/or qualitatively different effects on action preparation and performance. For instance, one could argue that a difference in the results might simply be due to an artifact in the methods used to induce the two types of expectation. On the one hand, reading aloud the cues does not enforce deep processing. In an implicit sequence learning study with a repeating sequence of to be read words, Hartman et al. (1989) demonstrated a surprising lack of explicit and even implicit learning. Generating the predictions, on the other hand, might enforce deeper processing for various reasons. For instance, participants were instructed that expectations should not be the same all the time. The experimenter was present outside the EEG booth coding the expectations online. Thus, the self-generated expectations were constrained such that they should be somewhat variable from trial-to-trial, avoiding perseverance and obvious patterns. This likely enforced that participants allocated a substantial part of their resources to the expectations in the prediction version of the task (compare e.g., Rapoport and Budescu, 1997).

Looking for functional differences between different types of expectation, Bubic et al. (2009, 2010) employed EEG and fMRI to investigate involved brain structures and processes. Violations of sequential regularities were accompanied by increased activity in premotor and cerebellar components of the “sequencing network,” presumably reflecting a mismatch between expectations generated by a forward model (cf. Wolpert and Ghahramani, 2000) and the observed stimuli – and an adjustment of the model. In addition, lateral prefrontal areas were engaged when a sequence violation required a boost in cognitive control. Stimuli deviating from a context of standard stimuli by a certain feature (as in an oddball paradigm), on the other hand, triggered responses in bilateral posterior temporal and parietal areas, reflecting increased attention and perceptual processing (Bubic et al., 2009). Interestingly, they also report differences in both the N2 and P3 components between their expectation conditions. While the N2 exhibited a shorter latency for sequential deviants compared to feature deviants, the P3 peaked later in the first condition and had a smaller amplitude. In line with the activation pattern reported in their imaging study, both components had a more posterior distribution for feature deviants. Additionally, they identified an enhanced N1 component for feature deviants, suggesting an early sensory registration of the irregularity (Bubic et al., 2010). The authors take these findings as indication for distinct functional networks involved in the processing of different types of expectation. It remains an interesting question whether similar functional differences also apply to the distinction between self-generated and externally cued expectation studied here.

## Conclusion

Self-generated expectations differ from cue-induced expectations on a range of cognitive processing stages and result in stronger behavioral effects. Response time benefits for expected stimuli are much larger when expectations are self-generated as compared to externally cued. Higher amplitudes in both the N2 and P3 components for violations of self-generated expectations indicate increased premotoric preparation compared to cue-induced expectations. This goes along with a missing benefit for stimuli matching the expected response only and is mirrored in the LRP. Underlying cognitive or neuronal functional differences between these types of expectation remain a subject for future studies.

## Conflict of Interest Statement

The authors declare that the research was conducted in the absence of any commercial or financial relationships that could be construed as a potential conflict of interest.

## Footnotes

^1^Voice onset time results from the behavioral pilot study; predictions: *M* = 467 ms (SD = 117.03), cues: *M* = 465 ms (SD = 49.05), *t*(9) = 0.08, ns.

^2^Number of trials, excluding errors: predictions: mismatch *M* = 257 (SD = 13); response match *M* = 127 (SD = 11); stimulus match *M* = 138 (SD = 11); cues: mismatch *M* = 268 (SD = 7); response match *M* = 119 (SD = 5); stimulus match *M* = 149 (SD = 4).

## References

[B1] Acosta E. (1982). Subjective and objective determinants of expectancy: similarities and differences. Am. J. Psychol. 95, 139–160 10.2307/1422664

[B2] Arrington C. M. Logan G. D. (2005). Voluntary task switching: chasing the elusive homunculus. J. Exp. Psychol. Learn. Mem. Cogn. 31, 683–702 10.1037/0278-7393.31.4.683 16060773

[B3] Astor-Jack T. Haggard P. (2005). “Intention and reactivity,” in Attention in Action: Advances from Cognitive Neuroscience, eds Humphreys G. W. Riddoch M. J. (Hove: Psychology Press), 109–130

[B4] Ayton P. Fischer I. (2004). The hot hand fallacy and the gambler’s fallacy: two faces of subjective randomness? Mem. Cognit. 32, 1369–1378 10.3758/BF03206327 15900930

[B5] Baldwin J. M. (1895). Types of reaction. Psychol. Rev. 2, 259–273 10.1037/h0074743

[B6] Ballard D. Hayhoe M. Pelz J. (1995). Memory representations in natural tasks. J. Cogn. Neurosci. 7, 66–80 10.1162/jocn.1995.7.1.66 23961754

[B7] Bargh J. A. Chartrand T. L. (1999). The unbearable automaticity of being. Am. Psychol. 54, 462–479 10.1037/0003-066X.54.7.462

[B8] Brainard D. H. (1997). The psychophysics toolbox. Spat. Vis. 10, 443–446 10.1163/156856897X00375 9176952

[B9] Bubic A. Bendixen A. Schubotz R. I. Jacobsen T. Schröger E. (2010). Differences in processing violations of sequential and feature regularities as revealed by visual event-related brain potentials. Brain Res. 1317, 192–202 10.1016/j.brainres.2009.12.072 20051231

[B10] Bubic A. von Cramon D. Y. Jacobsen T. Schröger E. Schubotz R. I. (2009). Violation of expectation: neural correlates reflect bases of prediction. J. Cogn. Neurosci. 21, 155–168 10.1162/jocn.2009.21013 18476761

[B11] Coles M. G. H. (1989). Modern mind brain reading: psychophysiological, physiology, and cognition. Psychophysiology 26, 251–269 10.1111/j.1469-8986.1989.tb01916.x 2667018

[B12] Damen E. J. P. Brunia C. H. M. (1994). Is a stimulus conveying task-relevant information a sufficient condition to elicit a stimulus-preceding negativity? Psychophysiology 31, 129–139 10.1111/j.1469-8986.1994.tb01033.x 8153249

[B13] Dreisbach G. Haider H. (2008). That’s what task sets are for: shielding against irrelevant information. Psychol. Res. 72, 355–361 10.1007/s00426-007-0131-5 18057961

[B14] Elsner B. Hommel B. (2001). Effect anticipation and action control. J. Exp. Psychol. Hum. Percept. Perform. 27, 229–240 10.1037/0096-1523.27.1.229 11248937

[B15] Fabiani M. Gratton G. Karis D. Donchin E. (1987). “The definition, identification, and reliability of measurement of the P300 component of the event-related brain potential,” in *Advances in Psychophysiology*, Vol. 2, eds Ackles P. K. Jennings J. R. Coles M. G. H. (Greenwich, CT: JAI Press), 1–78

[B16] Fan J. Kolster R. Ghajar J. Sh M. Knight R. T. Sarkar R. (2007). Response anticipation and response conflict: an event-related potential and functional magnetic resonance imaging study. J. Neurosci. 27, 2272–2282 10.1523/JNEUROSCI.1833-07.2007 17329424 PMC6673473

[B17] Fitts P. M. Peterson J. R. Wolpe G. (1963). Cognitive aspects of information processing: II. Adjustments to stimulus redundancy. J. Exp. Psychol. 65, 423–432 10.1037/h0047993 13945344

[B18] Folstein J. R. Van Petten C. (2008). Influence of cognitive control and mismatch on the N2 component of the ERP: a review. Psychophysiology 45, 152–170 10.1111/j.1469-8986.2007.00628.x 17850238 PMC2365910

[B19] Hackley S. A. Valle-Inclán F. (1998). Automatic alerting does not speed late motoric processes in a reaction-time task. Nature 291, 786–788 10.1038/35849 9486647

[B20] Hartman M. Knopman D. S. Nissen M. J. (1989). Implicit learning of new verbal associations. J. Exp. Psychol. Learn. Mem. Cogn. 15, 1070–1082 10.1037/0278-7393.15.6.1070 2530307

[B21] Herwig A. Prinz W. Waszak F. (2007). Two modes of sensorimotor integration in intention-based and stimulus-based actions. Q. J. Exp. Psychol. 60, 1540–1554 10.1080/17470210601119134 17853217

[B22] Herwig A. Waszak F. (2012). Action-effect bindings and ideomotor learning in intention- and stimulus-based actions. Front. Psychol. 3:444 10.3389/fpsyg.2012.00444 23112785 PMC3481004

[B23] Hommel B. (2009). Action control according to TEC (theory of event coding). Psychol. Res. 73, 512–526 10.1007/s00426-008-0186-y 19337749 PMC2694931

[B24] Jahanshahi M. Saleem T. Ho A. K. Dirnberger G. Fuller R. (2006). Random number generation as an index of controlled processing. Neuropsychology 20, 391–399 10.1037/0894-4105.20.4.391 16846257

[B25] Janczyk M. Heinemann A. Pfister R. (2012). Instant attraction: immediate action-effect bindings occur for both, stimulus- and goal-driven actions. Front. Psychol. 3:446 10.3389/fpsyg.2012.00446 23112787 PMC3481005

[B26] Jarmasz J. Hollands J. G. (2009). Confidence intervals in repeated-measures designs: the number of observations principle. Can. J. Exp. Psychol. 63, 124–138 19485604 10.1037/a0014164

[B27] Jentzsch I. Sommer W. (2002). The effect of intentional expectancy on mental processing: a chronopsychophysiological investigation. Acta Psychol. (Amst.) 111, 265–282 10.1016/S0001-6918(02)00053-7 12422949

[B28] Jiménez L. Méndez A. (2012). It is not what you expect: dissociating conflict adaptation from expectancies in a Stroop task. J. Exp. Psychol. Hum. Percept. Perform. [Epub ahead of print]. 10.1037/a0027734 22428671

[B29] Kahneman D. Tversky A. (1982). Variants of uncertainty. Cognition 11, 143–157 10.1016/0010-0277(82)90022-1 7198958

[B30] Kopp B. Rist F. Mattler U. (1996). N200 in the flanker task as a neurobehavioral tool for investigating executive control. Psychophysiology 33, 282–294 10.1111/j.1469-8986.1996.tb00425.x 8936397

[B31] Kunde W. (2001). Response-effect compatibility in manual choice reaction tasks. J. Exp. Psychol. Hum. Percept. Perform. 27, 387–394 10.1037/0096-1523.27.2.387 11318054

[B32] Kunde W. (2004). Response priming by supraliminal and subliminal action effects. Psychol. Res. 68, 91–96 10.1007/s00426-003-0156-3 14634809

[B33] Kunde W. Elsner K. Kiesel A. (2007). No anticipation – no action. The role of anticipation in action and perception. Cogn. Process. 8, 71–78 10.1007/s10339-007-0162-2 17340106

[B34] Leuthold H. Sommer W. Ulrich R. (1996). Partial advance information and response preparation: inferences from the lateralized readiness potential. J. Exp. Psychol. Gen. 125, 307–323 10.1037/0096-3445.125.3.307 8830109

[B35] Li L. Wang M. Zhao Q.-J. Fogelson N. (2012). Neural mechanisms underlying the cost of task switching: an ERP study. PLoS ONE 7:e42233 10.1371/journal.pone.0042233 22860090 PMC3408496

[B36] Loftus G. R. Masson M. E. J. (1994). Using confidence intervals in within-subject designs. Psychon. Bull. Rev. 1, 476–490 10.3758/BF03210951 24203555

[B37] Magen H. Cohen A. (2010). Modularity beyond perception: evidence from the PRP paradigm. J. Exp. Psychol. Hum. Percept. Perform. 36, 395–414 10.1037/a0017174 20364926

[B38] Matt J. Leuthold H. Sommer W. (1992). Differential effects of voluntary expectancies on reaction times and event-related potentials: evidence for automatic and controlled expectancies. J. Exp. Psychol. Learn. Mem. Cogn. 18, 810–822 10.1037/0278-7393.18.4.810 1385618

[B39] Miller J. Patterson T. Ulrich R. (1998). Jackknife-based method for measuring LRP onset latency differences. Psychophysiology 35, 99–115 10.1111/1469-8986.3510099 9499711

[B40] Nattkemper D. Ziessler M. Frensch P. A. (2010). Binding in voluntary action control. Neurosci. Biobehav. Rev. 34, 1092–1101 10.1016/j.neubiorev.2009.12.013 20036685

[B41] Neuringer A. Jensen G. (2010). Operant variability and voluntary action. Psychol. Rev. 117, 972–993 10.1037/a0019499 20658860

[B42] Nicholson R. Karayanidis F. Poboka D. Heathcote A. Michie P. T. (2005). Electrophysiological correlates of anticipatory task-switching processes. Psychophysiology 42, 540–554 16176376 10.1111/j.1469-8986.2005.00350.x

[B43] Nigbur R. Ivanova G. Stürmer B. (2011). Theta power as a marker for cognitive interference. Clin. Neurophysiol. 122, 2185–2194 10.1016/j.clinph.2011.03.030 21550845

[B44] Oswal A. Ogden M. Carpenter R. H. (2007). The time course of stimulus expectation in a saccadic decision task. J. Neurophysiol. 97, 2722–2730 10.1152/jn.01238.2006 17267751

[B45] Patel S. H. Azzam P. N. (2005). Characterization of N200 and P300: selected studies of the event-related potential. Int. J. Med. Sci. 2, 147–154 16239953 10.7150/ijms.2.147 PMC1252727

[B46] Pelli D. G. (1997). The videotoolbox software for visual psychophysics: transforming numbers into movies. Spat. Vis. 10, 437–442 10.1163/156856897X00366 9176953

[B47] Pfister R. Heinemann A. Kiesel A. Thomaschke R. Janczyk M. (2012). Do endogenous and exogenous action control compete for perception? J. Exp. Psychol. Hum. Percept. Perform. 38, 279–284 10.1037/a0026658 22201462

[B48] Pfister R. Kiesel A. Hoffmann J. (2011). Learning at any rate: action-effect learning for stimulus-based actions. Psychol. Res. 75, 61–65 10.1007/s00426-010-0288-1 20490862

[B49] Pfister R. Kiesel A. Melcher T. (2010). Adaptive control of ideomotor effect anticipations. Acta Psychol. (Amst.) 135, 316–322 10.1016/j.actpsy.2010.08.006 20875631

[B50] Posner M. I. Snyder C. R. R. (1975). “Facilitation and inhibition in the processing of signals,” in Attention and Performance V, eds Rabbitt P. M. A. Domic S. (New York: Academic Press), 669–682

[B51] Rapoport A. Budescu D. V. (1997). Randomization in individual choice behavior. Psychol. Rev. 104, 603–617 10.1037/0033-295X.104.3.603

[B52] Scheibe C. Schubert R. Sommer W. Heekeren H. R. (2009). Electrophysiological evidence for the effect of prior probability on response preparation. Psychophysiology 46, 758–770 10.1111/j.1469-8986.2009.00825.x 19490511

[B53] Shin Y. K. Proctor R. W. Capaldi E. J. (2010). A review of contemporary ideomotor theory. Psychol. Bull. 136, 943–974 10.1037/a0021628 20822210

[B54] Shulman G. L. Ollinger J. M. Akbudak E. Conturo T. E. Snyder A. Z. Petersen S. E. (1999). Areas involved in encoding and applying directional expectations to moving objects. J. Neurosci. 19, 9480–9496 10531451 10.1523/JNEUROSCI.19-21-09480.1999 PMC6782891

[B55] Sutton R. S. Barto A. G. (1981). Toward a modern theory of adaptive networks: expectation and prediction. Psychol. Rev. 88, 135–170 10.1037/0033-295X.88.2.135 7291377

[B56] Sutton R. S. Tueting P. Zubin J. John E. R. (1967). Information delivery and the sensory evoked potential. Science 155, 1436–1439 10.1126/science.155.3768.1436 6018511

[B57] Tillman C. M. Wiens S. (2011). Behavioral and ERP indices of response conflict in Stroop and flanker tasks. Psychophysiology 48, 1405–1411 10.1111/j.1469-8986.2011.01203.x 21457276

[B58] Titchener E. B. (1895). The type-theory of simple reaction. Mind 4, 506–514 10.1093/mind/IV.16.506

[B59] Ulrich R. Miller J. (2001). Using the jackknife-based scoring method for measuring LRP onset effects in factorial designs. Psychophysiology 38, 816–827 10.1111/1469-8986.3850816 11577905

[B60] Umbach V. J. Schwager S. Frensch P. A. Gaschler R. (2012). Does explicit expectation really affect preparation? Front. Psychol. 3:378 10.3389/fpsyg.2012.00378 23248606 PMC3521289

[B61] Wenke D. Gaschler R. Nattkemper D. (2007). Instruction-induced feature binding. Psychol. Res. 71, 92–106 10.1007/s00426-005-0038-y 16341902

[B62] Wenke D. Gaschler R. Nattkemper D. Frensch P. A. (2009). Strategic influences on implementing instructions for future actions. Psychol. Res. 73, 587–601 10.1007/s00426-009-0240-4 19360437 PMC2694933

[B63] Wolpert D. Ghahramani Z. (2000). Computational principles of movement neuroscience. Nat. Neurosci. 3, 1212–1217 10.1038/81497 11127840

[B64] Zießler M. Nattkemper D. (2002). “Effect anticipation and action planning,” in Common Mechanisms in Perception and Action: Attention and Performance XIX, eds Prinz W. Hommel B. (Oxford: Oxford University Press), 645–672

